# Targeting Ovarian Neoplasms: Subtypes and Therapeutic Options

**DOI:** 10.3390/medicina61122246

**Published:** 2025-12-18

**Authors:** Seon Young Hong, Ahyoung Cho, Chang-Suk Chae, Hye Jin You

**Affiliations:** 1 Department of Cancer Biomedical Science, National Cancer Center Graduate School of Cancer Science and Policy, National Cancer Center, Goyang 10408, Republic of Korea; 97199@ncc.re.kr; 2 Cancer Microenvironment Branch, Division of Cancer Biology, Research Institute, National Cancer Center, Goyang 10408, Republic of Korea

**Keywords:** ovarian cancer, genetic alteration, tumor microenvironment, lipid mediators

## Abstract

The ovary, as the primary organ responsible for reproduction and new life, plays a central role in female development, maturation, and health. Neoplasms arising from the ovary and its associated tissues exhibit substantial heterogeneity in their histopathological and molecular profiles, many of which remain poorly understood. This review aims to summarize recent advances in the understanding of genetic alterations underlying ovarian neoplasms and to explore therapeutic strategies informed by molecular biomarkers and tumor microenvironmental factors. A comprehensive literature search was performed, focusing on genomic alterations, biomarker-guided therapies, and tumor microenvironmental modulation in ovarian cancers. Emphasis was placed on studies addressing lipid mediator pathways and their roles in immune regulation and therapeutic response. Based on diagnostic classifications, recurrent alterations in *TP53*, *MYC*, *PIK3CA*, and *KRAS* are consistently observed across epithelial and germ cell ovarian tumors, whereas non-epithelial subtypes such as sex cord–stromal tumors (SCSTs) and small-cell carcinoma of the ovary, hypercalcemic type (SCCOHT), are predominantly associated with *ARID1A* and *SMARCA4* mutations, respectively. These findings highlight distinct pathogenic mechanisms linked to specific genetic alterations and reveal potential therapeutic vulnerabilities. Moreover, lipid metabolism has been closely implicated in immune surveillance through STING signaling cascades within innate immune cells, suggesting that lipid mediators and their associated genes may represent promising therapeutic targets in ovarian cancers (OCs). Targeting lipid mediators could be particularly effective in relapsed OCs, as modulating innate immune cells within the tumor microenvironment (TME) may enhance immune surveillance and improve antitumor responses. Integrating genetic and microenvironmental insights offers a promising direction for developing more effective and personalized therapeutic strategies in OC.

## 1. Introduction

According to recent statistics, 55% of ovarian cancer (OC) patients in the United States are diagnosed with distant metastasis, resulting in a 5-year survival rate of only 31.8% [1]. Similarly, in South Korea, 44.5% of OC patients present with distant metastasis, with a corresponding 5-year survival rate of 44.4% [2,3]. In contrast, patients diagnosed with localized (~90%) or regional (~80%) OC have significantly higher survival rates, highlighting the critical need to understand the tumor microenvironment (TME) in order to improve prognosis in metastatic cases.

Recent technological advancements have enabled a deeper understanding of tumorigenesis, malignancy, and drug resistance at the molecular level. These insights have led to the development of advanced therapeutic strategies, many of which are now being evaluated in clinical trials. However, most of the available data—particularly from multiomics-based approaches—derive from large cohorts, predominantly composed of epithelial OCs (EOCs), leaving other ovarian and related neoplasms underrepresented. Moreover, effective therapeutic options for patients with metastatic disease remain limited.

For this review, we collected literature related to OC subtypes—including pathology, diagnostic features, and genetic alterations, without imposing year limitations—from the 2020 WHO classification, cBioPortal databases, and PubMed. When multiple articles addressed the same subtype, we prioritized more recent publications and, when applicable, studies with larger patient cohorts for citation.

Based on this information, we provide an overview of neoplasms arising from ovarian and related tissues, highlighting the associated genetic markers and their relevance to therapeutic strategies. We also examine key components of the TME that influence immune surveillance, with a particular focus on lipid mediators and related immune cells, to inform future therapeutic applications.

## 2. OC—Histological Subtypes and Genetic Alterations

### 2.1. OC—Histological Subtypes

Based on histological classification, neoplasms originating from the ovary and related tissues are categorized into seven major types according to their tissue of origin: epithelial (E), mesenchymal, mixed epithelial and mesenchymal, sex cord–stromal (SCS), germ cell (GC), miscellaneous tumors, and tumor-like lesions (Table 1). Within each category, tumors are further classified by their histological characteristics and degree of malignancy—benign, borderline, or malignant (carcinoma). Epithelial ovarian carcinomas (EOCs), the most common subtype, are further subclassified based on histological and prognostic features into high-grade serous (HGS), low-grade serous (LGS), mucinous (M), endometrioid (E), clear cell (CC), Brenner (B), mesonephric-like, undifferentiated, carcinosarcoma, and mixed carcinomas, as outlined by the World Health Organization (WHO) classification of female genital tumors over the past five decades [4,5,6]. These carcinoma subtypes exhibit significant variability in clinical features and molecular composition, often more so than in cell of origin, leading to distinct therapeutic approaches [7].

In South Korea, epithelial carcinomas comprise approximately 81% of all OCs [2]. Among these, serous carcinoma (SOC) is the most prevalent (44.9%), followed by clear cell carcinoma (CCC, 10.2%), endometrioid carcinoma (EndOC, 9.4%), mucinous carcinoma (MOC, 8.8%), and adenocarcinoma not otherwise specified (NOS, 2.9%) [2,3,8]. Non-epithelial OCs include sex cord–stromal tumors (SCSTs, 5.5%) and germ cell tumors (GCTs, 4.2%). Recent advancements in epidemiologic analyses have enabled more refined identification of OC risk factors [7].

Most EOCs show a 5-year survival rate of 80–100% when localized, and 70–90% when regionally spread [9]. However, survival drops significantly to a variable 16–60% when the cancer has metastasized distantly [9]. Specifically in the United States, the 5-year survival rates for HGSOC, CCC, and MOC are 34.5%, 24.0%, and 16.6%, respectively [10]. Similarly, in Norway, 5-year survival rates for advanced-stage (Stage III–IV) HGSOC and CCC are reported to be under 46% and 31%, respectively [11], underscoring the need for robust preclinical models for advanced OC to inform therapeutic strategies. Furthermore, OCs are usually diagnosed with peritoneal metastasis, so that TME is critical for multifocal therapeutic strategies. Non-epithelial ovarian tumors—comprising SCSTs, GCTs, miscellaneous tumors, and tumor-like lesions—are also listed in Table 1. Among these, SCSTs and GCTs are the most prominent subtypes, though their incidence remains low, estimated at 2.1–3.7 cases per million women. Malignant GCTs are responsible for approximately 80% of malignant ovarian tumors in preadolescent females, whereas SCSTs occur across a broader age range, accounting for up to 5% of ovarian malignancies [12,13]. According to a cohort study conducted across MITO centers, the 5-year survival rates for GCT subtypes were as follows: 100% for immature teratomas, 97.9% for dysgerminomas, 69.9% for yolk sac tumors, and 62.9% for mixed germ cell tumors [14].

**Table 1:** Neoplasms from ovary and related tissues and markers associated with molecular events or diagnosis.

Tumor Types (WHO 2020 Classification)	Associated with	Biomarkers for Diagnosis	Ref.
1. Epithelial 1.1. Serous Serous cystadenoma, adenofibroma and surface papilloma Serous borderline tumor Low-grade serous carcinoma High-grade serous carcinoma	Polyclonal alteration of KRAS, BRAF Copy number alterations, BRAF, KRAS KRAS, BRAF, NRAS, and CDKN2A; gain of 1q or 18p; loss of 1p, 18q, and 22 *TP53*, *PIK3CA*, *HLTF*, *POLQ*, *PIK3CB*, *MET*, *ARID1B*, *NF1*, *MRE11A*, *CCNE1*, *RB1*, *CDK12*, *PTEN*, *TP53BP1*, *BRCA1*, *BRCA2*. Homologous recombination RNA repair defects, whole-genome duplication, MHC-II expression	WT1, estrogen receptor	[4,15,16,17,18,19,20,21,22,23,24,25,26,27,28,29,30]
			[31,32,33,34]
1.2. Mucinous Mucinous cystadenoma and adenofibroma Mucinous borderline tumor Mucinous carcinoma 1.3. Endometrioid tumors Endometrioid cystadenoma and adenofibroma Endometrioid borderline tumor Endometrioid carcinoma 1.4. Clear cell tumors Clear cell cystadenoma and adenofibroma Clear cell borderline tumor Clear cell carcinoma 1.5. Seromucinous tumors Seromucinous cystadenoma and adenofibroma Seromucinous borderline tumor 1.6. Brenner tumors Brenner tumor Borderline Brenner tumor Malignant Brenner tumor 1.7. Other carcinomas Mesonephric-like adenocarcinoma Undifferentiated and dedifferentiated carcinoma Carcinosarcoma Mixed carcinoma	*ARID1A* *TP53*, *RNF43*, *ELF3*, *GNAS*, *ERBB3*, *KLF5*		
	*ARID1A*, *CTNNB1*, *CTNNB1*, *PTEN*, *POLE*, *MSI*,	AKT	[5,35,36,37,38,39,40]
	*ARID1A* *ARID1A*, *PTEN*, *PIK3CA*, *TP53*		[40,41,42,43,44,45,46,47,48,49,50]
	*ARID1A* *MDM2*, *PIK3CA* *NRAS*, *KRAS*; gain of 1q or 18p; loss of 1p, 18q, and 22	GATA3, TTF1, CD10	[34]
			[10,32,33,51,52]
	*KIT*, *EGFR*, *HER2*, *TP53*, *PTEN*, *CHD4*, *BCOR*, *KRAS*, *PIK3CA*, *ARID1A*, *CTNNB1*		[17,26,36,53,54,55,56,57,58,59,60,61]
2. Mesenchymal 2.1. Endometrioid stromal sarcoma Low-grade endometrioid stromal sarcoma High-grade endometrioid stromal sarcoma 2.2. Smooth muscle tumors Leiomyoma Smooth muscle tumor of uncertain malignant potential Leiomyosarcoma 2.3. Ovarian myxoma	*JAZF1::SUZ12* fusion	CD10	[54,62,63]
	*YWHAE::FAM22* fusion	CD10	
		CD10	
3. Mixed epithelial and mesenchymal tumors Adenosarcoma		CD10	[54,62]
4. Sex cord–stromal tumors 4.1. Pure stromal tumors Fibroma Thecoma Sclerosing stromal tumor Microcystic stromal tumor Signet ring stromal tumor Leydig cell tumor Steroid cell tumor Fibrosarcoma 4.2. Pure sex cord tumors Adult granulosa cell tumor Juvenile granulosa cell tumor Sertoli cell tumor Sex cord tumor with annular tubules 4.3. Mixed sex cord–stromal tumors Sertoli–Leydig cell tumor Sex cord–stromal tumor, NOS Gynandroblastoma	*FHL2::GLI2* fusion *CTNNB1*, *APC* —		[64,65,66,67,68,69,70,71,72,73,74,75,76,77,78,79,80]
	*FOXL2* *AKT1*		
	*STK11*		
	*DICER1*, *FOXL2*		
	*DICER1*		
5. Germ cell tumors 5.1. Teratoma, benign 5.2. Immature teratoma, NOS 5.3. Dysgerminoma 5.4. Yolk sac tumor 5.5. Embryonal carcinoma	Isochromosome 12p Isochromosome 12p, *KIT* Isochromosome 12p Isochromosome 12p	AFP Sall4, OCT3/4, LDH Sall4, AFP Sall4, OCT3/4, SOX2, β-hCG, AFP β-hCG LDH, AFP, β-hCG	[12,13,69,72,81,82,83,84,85,86,87,88,89,90,91,92]
5.6. Choriocarcinoma, NOS 5.7. Mixed germ cell tumor 5.8. Monodermal teratomas and somatic type tumors arising from a dermoid cyst Struma ovarii, NOS Struma ovarii, malignant Strumal carcinoid Teratoma with malignant transformation Cystic teratoma, NOS 5.9. Germ cell sex cord–stromal tumors Gonadoblastoma, Mixed germ cell—sex cord–stromal tumor, unclassified	*BRAF*	PLAP, OCT4	
	OCT3/4 12p amplification		
6. Miscellaneous tumors Rete cystadenoma, adenoma and adenocarcinoma Wolffian tumor Solid pseudopapillary tumor Small-cell carcinoma of the ovary, hypercalcemic type (SCCOHT) Wilms tumor	*CTNNB1* *SMARCA4*		[45,93,94,95,96,97,98,99,100,101,102,103]
7. Tumor-like lesions Follicle cyst Corpus luteum cyst Large solitary luteinized follicle cyst Hyperreactio luteinalis			[64,104]

NOS, not otherwise specified; AFP, α-fetoprotein; LDH, lactate dehydrogenase; β-hCG, beta-human chorionic gonadotropin.

### 2.2. OC-Genetic Alterations Associated with Subtypes

Several genes involved in key molecular events during OC development have been identified, many of which also represent potential targets for therapeutic intervention (Table 1). Subtype-specific genetic alterations were identified and further analyzed in terms of their alteration frequencies across several cohort studies, including those conducted by The Cancer Genome Atlas (TCGA) Research Network [29,105], the Memorial Sloan Kettering Cancer Center (MSKCC) [106,107], and others, as shown in Table 2 [31,97,108]. As expected, *TP53* is the most frequently altered gene in HGSOC, with mutations observed in over 90% of cases in each cohort [29,108]. Notably, *TP53* alterations are also present in approximately 50% of MOCs [31]. In contrast, LGSOC is characterized by a distinct pattern, with frequent *KRAS* mutations but a relatively low frequency of *TP53* alterations [107]. Data from one of the largest tissue-based cancer patient cohorts, the MSKCC-IMPACT database, include information on GCTs, such as mixed germ cell tumors, yolk sac tumors, choriocarcinomas, teratomas (both mature and immature), and teratomas with malignant transformation. In these tumors, mutation frequencies were reported as follows: *TP53* (54%), *KRAS* (28%), and *PTEN* (11%) [106]. Interestingly, in one of the miscellaneous tumor types—small-cell carcinoma of the ovary, hypercalcemic type (SCCOHT)—a striking dependence on *SMARCA4* alterations has been observed, with up to 92% of cases [97]. SCSTs were associated with *FOXL2* and *CTNNB1*, 68% and 16%, respectively.

## 3. OC—Current Therapeutic Strategies

Alterations in *TP53*, *MYC*, *PIK3CA*, and *KRAS* predominated in epithelial and GCTs, whereas *ARID1A* and *SMARCA4* mutations were characteristic of SCST and SCCOHT, respectively, indicating chromosomal instability in non-epithelial OCs (Table 2). Alterations in *BRCA1* are frequently observed in OCs (Table 2). A recent study identified an intriguing association between BRCA1 mutation carriers and elevated blood molybdenum levels [109], a trace element linked to mitochondrial biogenesis [110]. However, the majority of genetic alterations are still being investigated for their roles as risk factors, and effective therapeutic strategies to target these alterations remain in early stages of development. Regardless of genetic alterations, standard treatments against OCs include cytoreductive surgery and chemotherapy with platinum drugs. Despite initial responsiveness to cytoreductive surgery and platinum-based chemotherapy, the majority of patients with ovarian cancer ultimately experience disease relapse. Approximately 70% of patients develop recurrent, more aggressive, and chemotherapy-resistant tumors following first-line therapy [111]. Conventional treatment strategies—comprising maximal cytoreductive surgery followed by adjuvant chemotherapy—have succussed only modest improvements over the past five decades, and the 5-year survival rate for patients with advanced or metastatic disease remains below 30% [112]. Even among those receiving optimal standard-of-care regimens, most relapse within a few years, and subsequent lines of therapy yield diminishing responses, culminating in a 5-year overall survival rate of only 30–40% worldwide. The introduction of maintenance therapy with poly(ADP-ribose) polymerase inhibitors (PARPis) has extended progression-free survival (PFS) and, in selected populations, improved long-term overall survival [113,114]. Nevertheless, a substantial proportion of patients either fail to respond initially or acquire resistance during treatment, limiting durable benefit. Such intrinsic and acquired drug resistance remains a major therapeutic obstacle and the principal cause of poor prognosis in ovarian cancer. Overcoming these resistance mechanisms (spanning defects in DNA damage response reprogramming, replication stress tolerance, and TME-mediated immune suppression) represents a critical unmet need for improving patient outcomes.

## 4. OC—Therapeutic Strategies Targeting TME

### 4.1. Lipid-Driven Immune Suppression in the Ovarian Cancer TME

#### 4.1.1. Tumor-Intrinsic Lipid-Mediated Immunosuppression

Aberrant lipid metabolism and bioactive lipid mediators are increasingly recognized as key determinants of immune evasion in ovarian cancer. Cyclooxygenase-derived prostaglandin E_2_ (PGE_2_), mainly produced in tumors, is a central lipid mediator in tumors and directly disables dendritic cell (DC) function via EP2/EP4 signaling, skewing myeloid cells toward suppressive phenotypes and blunting T-cell priming. Selective EP2/EP4 blockade restores antitumor DC activity in human settings, underscoring the pathway’s translational relevance [115]. Beyond PGE_2_, arachidonate 5-lipoxygenase (5-LOX)-derived leukotrienes correlate with poor prognosis, enhance invasion, and recruit tumor-promoting macrophages, reinforcing a lipid driven immunosuppressive loop. COX-2/PGE_2_ signaling also promotes VEGF and metastatic programs that indirectly suppress immune control [116].

#### 4.1.2. Tumor-Extrinsic Lipid-Mediated Immunosuppression

Ovarian tumors grow in adipose-rich peritoneal/omental spaces. Adipocyte-derived lipid moieties make tumors resistant to immune-activating oncolytic virotherapy. Depleting lipids from adipocyte-conditioned medium or blocking fatty acid uptake resensitizes OC models, highlighting how excess fatty acids can keep “cold” tumors. At a tissue scale, high-fat environments reduce intratumoral viral titers and favor therapeutic resistance [117].

Ascites and omental niches provide abundant fatty acids that OC cells rapidly absorb, boosting ATP production, suppressing AMPK, and activating mTOR/TAK1–NF-κB programs that drive aggressiveness that also favor immunosuppression. Targeting this axis (AMPK activation + TAK1 and FASN inhibition) synergistically impairs peritoneal metastases, suggesting a route to relieve lipid conditioned immune dysfunction [118]. OC-derived TGF-β1 converts omental adipocytes into cancer-associate adipocytes (CAAs) through SMAD3/TRIB3, which remodel the extracellular matrix (ECM, collagen/fibronectin) and raise IL-1β/IL-6 changes that support implantation and can further skew myeloid cells toward suppressive states. Pharmacologic blockade of TGF-β/SMAD3 prevents CAA/PMN formation and reduces metastatic burden [119].

### 4.2. Type I Interferon (IFN) and STING Activation Pathways in OC: Mechanistic Insights and Therapeutic Opportunities

The cGAS–STING pathway acts as a cytosolic DNA-sensing system that connects innate detection to adaptive immune activation. Binding of cytoplasmic DNA activates cGAS, generating cyclic GMP–AMP (cGAMP) that engages STING, leading to TBK1–IRF3 phosphorylation and type I interferon (IFN-I) production. These cytokines drive DC maturation, antigen presentation, and CD8^+^ T-cell priming. In OCs, persistent genomic instability continuously releases DNA into the cytosol, yet post-translational repression, ER stress, and lipid-mediated suppression often blunt STING signaling. A recent review of gynecologic malignancies reported that cGAS and STING are broadly expressed but functionally silenced in OC, indicating that pharmacologic re-engagement of this pathway could restore antitumor immunity [120]. DNA damage-response (DDR) inhibitors can activate cGAS–STING by generating cytosolic DNA. In BRCA1-deficient OC, PARP inhibition provokes STING-dependent IFN-β secretion, CD8^+^ T-cell infiltration, and tumor regression; loss of STING or TBK1 abolishes these effects [121]. Moreover, PARP inhibition upregulates PD-L1, CXCL10, and CCL5, explaining synergy with PD-L1 blockade [122]. Similarly, CX-5461, an RNA polymerase I inhibitor, causes nucleolar stress and cytosolic-DNA accumulation that activates STING–IRF3 signaling and type I IFN transcription, suppressing tumor growth [123]. Together, DDR-targeting agents transform immune-cold OC into IFN-rich, T-cell-inflamed microenvironments.

Ovarian tumors employ intrinsic brakes on STING activation. The deubiquitinase, USP35, binds phosphorylated STING and removes activating K63-linked ubiquitin chains, thereby inhibiting TBK1 and IRF3 phosphorylation and suppressing IFN-β expression [124]. Similarly, mitotic disruption can reignite innate sensing: CENPM knockdown triggers cytosolic-DNA accumulation, activates cGAS–STING, and induces pyroptosis, suppressing ovarian-tumor growth [125]. Relieving post-translational inhibition or exploiting mitotic stress thus restores endogenous STING–IFN signaling in OC.

Cofactor ions and metabolic states modulate STING activation. Manganese (Mn^2+^), a natural cGAS cofactor, enhances enzymatic activity. In the preclinical OC model, Mn^2+^ strengthened macrophage phagocytosis, CD8^+^ recruitment, and type I IFN production, reshaping the tumor milieu toward immune stimulation [126]. At the adaptive level, raising T-cell NAD^+^ downregulates the Golgi transporter SURF4, stabilizing STING. This NAD^+^–SURF4–STING axis, discovered by Jiacheng Shen et al. [127], enhances T-cell cytotoxicity and cooperates with tumor-cell STING activation. Hence, ionic and metabolic cues can globally reinforce STING-driven antitumor immunity [127].

Merging DDR targeting with nanotechnology can intensify STING activation. Polymer–PARP-inhibitor conjugates co-delivering a USP1 inhibitor (USP1i), which increased DNA damage, caused cytosolic-DNA accumulation, and robustly activated cGAS–STING signaling. This approach elevated IFN-I genes; enhanced CD8^+^ T-cell infiltration; and, when combined with PD-1 blockade, produced synergistic tumor regression in high-grade serous OC [128]. Such nanoplatforms exemplify next-generation synthetic-lethality immunotherapies that couple DNA-repair inhibition with innate immune activation. Clinically, BRCA1/HRD-positive tumors exhibit strong DDR–STING coupling and respond favorably to PARPi plus PD-L1 blockade [121,122]. Biomarkers such as phospho-STING, ISG signatures (CXCL10 and CCL5), and USP35 expression may help select patients. Because sustained STING activity can pivot toward NF-κB-dominant inflammation and T-cell exhaustion, future strategies should emphasize transient, immunogenic activation combined with checkpoint or metabolic therapy to produce durable, well-tolerated IFN-driven responses.

### 4.3. DC-Based Immunotherapy and Reprogramming Strategies in OC

The ovarian TME is profoundly immunosuppressive, characterized by lipid mediators, cytokines, and metabolic stress that collectively impair DC maturation and antigen presentation. OCs recruit large numbers of immature myeloid progenitors that differentiate into dysfunctional tumor-associated DCs, driven by β-defensin/CCR6-dependent chemotaxis and VEGF-A-mediated pro-angiogenic programming. These tumor-infiltrating DCs exhibit high PD-L1, CD277 (BTN3A1) expression, and Arginase-1 activity, and they release immunoregulatory cytokines that collectively suppress T-cell priming. Metabolic and stress-response pathways further exacerbate this dysfunction. OC-derived PGE_2_ and TGF-β jointly upregulate PD-L1 and Arginase-1 activity in DCs, while ER stress-induced activation of XBP1 promotes lipid droplet accumulation and defective cross-presentation. Downregulation of miR-155, a key regulator of antigen-presenting capacity in DC, also contributes to the tolerogenic phenotype [129]. Consequently, OC-associated DCs act not as immune initiators but as facilitators of angiogenesis and immune tolerance.

Recent advances in single-cell and functional analyses have delineated how different DC subsets are selectively modulated in OC. cDC1s (CD103^+^/XCR1^+^) are the principal cross-presenting cells responsible for CD8^+^ T-cell priming; however, their abundance and type I interferon signaling are markedly reduced in ascitic and metastatic lesions. cDC2s (CD11b^+^/Sirpα^+^) retain plasticity but frequently acquire a suppressive or Th2-skewing profile under the influence of IL-6, VEGF, and lactic acid. Plasmacytoid DCs (pDCs), though capable of secreting large amounts of type I interferons, often become functionally exhausted in the ovarian TME, expressing high levels of IDO1, PD-L1, and ICOS-L that promote regulatory T-cell (Treg) expansion and correlate with poor prognosis. The lipid mediator PGE_2_ plays a dominant role in DC suppression by signaling through EP2 and EP4 receptors.

Fibrinogen-like protein 2 (FGL2) has emerged as a potent DC-suppressive factor within OC ascites. FGL2, primarily produced by tumor-infiltrating macrophages/monocytes and DCs, downregulates CD40 and CD86 on CD11^+^ DCs through FcγRIIB/III-mediated signaling. In Fgl2^−/−^ mice, ovarian tumors displayed enhanced infiltration of cDC1s, CD3^+^ T cells, and DNAM-1^+^ NK cells, and they induced expression of co-stimulatory markers (MHC-II, CD86^+^, and CD40^+^) in spleen, resulting in reduced tumor burden. These findings identify FGL2 as a non-canonical immune checkpoint that limits DC maturation and adaptive immune activation [130].

DC-based vaccines have long been pursued as a therapeutic modality for OC. Early clinical trials were conducted using monocyte-derived DCs (moDCs) pulsed with HER-2/neu, WT-1, MUC1, or tumor lysates [131]. Contemporary approaches are refining vaccine composition through improved antigen loading, maturation stimuli, and DC subset selection. Reviews by Caro and Zhang emphasize the superior antigen-presenting potential of cDC1s and the promise of fusion-cell and in vivo targeted DC vaccines to enhance cytotoxic T-cell responses [131,132].

A particularly innovative strategy was reported by Zhang, who developed a DC–tumor cell fusion membrane nano-vaccine (FCM-NP). The FCM-NPs, loaded with CpG-ODN adjuvant, promoted DC maturation, and elicited robust CD8^+^ T-cell responses in multiple OC models, effectively delaying tumor growth and abdominal metastasis [133]. Restoration of type I IFN signaling is a critical determinant of DC immunogenicity. Nanoparticles co-delivering Mn^2+^ and platinum were shown to activate the cGAS–STING pathway in cancer cells, and they enhanced maturation of DCs (CD80^+^ CD86^+^) for potent antitumor immunity in OC models [134]. When combined with DC-targeted strategies such as EP2/EP4 inhibition or fusion-cell vaccination, these approaches hold potential to overcome the immune-exclusion barriers of advanced disease.

Collectively, these studies establish a mechanistic linking lipid-mediated suppression (PGE_2_–EP2/EP4 axis), immune checkpoints (FGL2 and PD-L1), and metabolic stress (IRE1 alpha-XBP1 pathway) to DC dysfunction in OC. Therapeutic reprogramming of DCs—through nanoparticle-based EP2/EP4 blockade, FGL2 inhibition, STING activation, or next-generation DC vaccines—offers a strategy to convert the ovarian TME from an immunologically “cold” environment into a type I IFN-rich, DC-driven immunogenic niche capable of sustaining durable T-cell-mediated tumor control.

### 4.4. Tumor-Associated Macrophages (TAMs) in OC: Reprogramming Immunosuppressive Niches Toward Antitumor Immunity

TAMs represent the dominant myeloid population in OC ascites and metastatic implants. These cells exhibit remarkable phenotypic plasticity, polarizing between M1-like (pro-inflammatory) and M2-like (immunoregulatory) states depending on tumor-derived cues. HGSOCs are characterized by a predominance of M2-like macrophages expressing CD163, CD206, ARG1, and IL-10, which foster immune evasion, angiogenesis, and chemotherapy resistance. In contrast, M1-like macrophages—driven by IFN-γ, TNF-α, or STING activation—correlate with improved survival outcomes through antigen presentation and cytotoxic T-cell recruitment. A recent study reported that the tumor ECM directly educates macrophages toward a tissue-remodeling, immunoregulatory phenotype that recapitulates the transcriptional profile of TAMs in patient metastases [135]. Using a decellularized omental metastasis model preserving the ECM’s native structure, the authors found that infiltrating monocytes differentiated into macrophages expressing a M0/M2-like phenotype. These “matrix-educated macrophages (MAMs)” diminished T-cell activation, revealing that ECM composition alone is sufficient to drive TAM polarization toward immune suppression and tissue remodeling [135]. Targeting desmoplastic ECM components (e.g., COL11A1, FN1, VCAN, MXRA5, and SFRP2) can recondition these MAMs, enhancing antitumor immunity. Complement pathways critically shape macrophage polarization within the ovarian TME. Fang identified that loss of KLHDC8A in normal ovarian epithelial cells triggers C5a secretion, which acts through C5aR/p65 NF-κB signaling to polarize macrophages toward a pro-tumoral phenotype [136]. This axis was validated by the reversal of M2 polarization (CD206) upon NF-κB inhibition, demonstrating that C5a/C5aR blockade may restore immune surveillance. Complementary evidence shows that C5aR inhibition synergizes with CXCL9 upregulation to enhance CD8^+^ T-cell infiltration, linking complement control with adaptive immunity reactivation [137].

Macrophage checkpoint pathways represent another central barrier to antitumor immunity. Liu first showed that CD47, a “don’t eat me” signal overexpressed on OC cells, suppresses macrophage phagocytosis and correlates with poor prognosis [138]. CD47 knockdown or blockade with monoclonal antibodies enhanced macrophage infiltration, increased the phagocytic index, and prolonged survival in xenografted mice [138]. Building on this, Yang developed a bispecific antibody fusion protein that simultaneously targets CD47/SIRPα and CD24/Siglec-10 axes—dual “don’t eat me” checkpoints co-expressed in OCs [139]. In preclinical models, PPAB001 therapy promoted phagocytosis of human OC cell line and enhanced tumor regression in SK-OV-3 xenografts [139]. Furthermore, a phase I clinical trial of the bispecific Fc-fusion protein SL-172154, which simultaneously blocks CD47 and activates antigen-presenting cells (APCs), has been reported [140]. These findings define bispecific macrophage checkpoint inhibitors as a potent next-generation immunotherapy to reprogram TAMs toward antitumor activity. Chemoresistance in OC is partly sustained by macrophage-driven immunosuppression. Li uncovered that the circular RNA circITGB6 promotes cisplatin resistance by driving TAM polarization via a circITGB6–IGF2BP2–FGF9 RNA–protein complex, which stabilizes FGF9 mRNA [141]. Elevated circITGB6 correlated with high CD206^+^ macrophage infiltration, mediating immunosuppressive TME. Notably, FGF9 deletion or ASO-mediated circITGB6 silencing restored M1 markers (TNF-α and iNOS) and decreased M2 cytokines (IL-10 and ARG1) [141]. Additionally, ongoing clinical trials of immune cell-based therapy are listed in Table 3.

Advances in biomaterial immunotherapy have enabled macrophage-targeted reprogramming. VandenHeuvel et al. introduced a lipid nanoparticle (LNP)-based siRNA system that selectively delivers siSIRPα to TAMs, effectively silencing the CD47–SIRPα checkpoint [148]. This “macrophage checkpoint nano-immunotherapy” reversed carboplatin resistance, and decreased spheroid invasion in 3D OC models. By exploiting the natural phagocytic properties of macrophages, these LNPs represent a scalable strategy to restore innate immune clearance without systemic toxicity.

Host obesity increases TAM density, lowers the pro-to-anti-inflammatory macrophage ratio, and is associated with reduced chemotherapy responsiveness. Strategies that deplete or repolarize M2-biased TAMs may counteract obesity-linked immune suppression and improve outcomes [149].

Collectively, these studies delineate multifaceted TAM reprogramming mechanisms in OC—ranging from ECM-mediated education and complement signaling to checkpoint inhibition and RNA-mediated polarization. Therapeutic integration of ECM remodeling, checkpoint blockade (CD47/CD24 bispecific antibodies), and circITGB6–FGF9 axis suppression could synergize with DC or NK cell-activating platforms, driving a comprehensive myeloid rejuvenation within the ovarian TME.

A phase I clinical trial of a mesothelin–CD40-bispecific antibody has been conducted in solid tumors, but its efficacy in ovarian cancer was found to be limited [150]. In addition, a phase I trial employing the myeloid-targeting antibodies PY159 and PY314 was performed; despite the complementary strategies of PY159 (promoting antitumor immunity) and PY314 (depleting TAMs), the results were largely limited to disease stabilization [151]. These findings highlight the need to understand the complex immunosuppressive mechanisms of the OC TME and to develop strategies for TAM suppression and reprogramming.

### 4.5. Natural Killer (NK) Cells in OC: Dysfunction, Engineering, and Combination Strategies

Innate cytotoxic NK cells are potent effectors against EOC, yet their function is often curtailed by ascites-borne suppressive cues. High peritoneal TGF-β1 was strongly associated with ascites-induced NK dysfunction, and it reduced progression-free and overall survival. Pharmacologic blockade of TGF-β signaling partially restored NK proliferation and function ex vivo, nominating TGF-β1 as a dominant soluble inhibitor in HGEOC [152]. Cytokine-inducible regulators also rise within the ovarian milieu: CISH, a regulator contributing to exhaustion is enriched in both early and late stages, with CISH levels positively associated with IL-10 and ER-stress marker GRP78 in tumor tissues—linking chronic cytokine/stress exposure to NK exhaustion [153]. Beyond cytokines, tumor iron overload leads to an immunosuppressive peritoneal niche. In a preclinical animal model and patient-derived organoid system, the FDA-approved chelator deferiprone reprogrammed OC cells to produce type I IFN, NKG2D ligand-encoding genes (e.g., *Mult1*, *H60b*, and *Ulbp1*) and drive NK-dependent control of metastatic disease. Mechanistically, iron chelation triggered releasing mtDNA and DNA-damage responses, amplifying IFN signaling and inducing a DC-IL-15-NK axis that accumulated NK cells at tumor sites and improved survival [154]. Cytokine-induced memory-like (CIML) NK cells retain heightened activation after brief cytokine priming. Arming CIML NKs with a mesothelin (MSLN)-targeting CAR (membrane-proximal epitope) produced durable antitumor activity in EOC lines, resisted dysfunction in patient ascites, and prevented metastasis in xenografts—outperforming conventional CAR-NKs [155]. To enable off-the-shelf manufacturing, hESC-derived MSLN CAR-NK cells were generated, and they eliminated human ovarian tumor cells, underscoring feasibility for standardized CAR-NK products [156]. For clinical translation, non-invasive tracking is crucial. HER2-CAR-NK-92 cells co-expressing the human sodium-iodide symporter (NIS) were visualized by PET, while simultaneously reducing tumor burden and prolonging survival in a HER2^+^ ovarian model—demonstrating that human-derived reporter imaging can monitor NK persistence and biodistribution alongside efficacy [157].

Oncolytic platforms can both arm NK cells and reshape tumor immunogenicity. A vIL-2-encoding oncolytic adenovirus selectively boosted effector NK/CD8^+^ T cells without expanding Tregs in ex vivo human ovarian co-cultures and improved in vivo control of patient-derived xenografts when combined with adoptive allogeneic NK therapy [158]. These experimental findings align with broader synthesis indicating that combination therapy (oncolytic viruses, targeted antibodies, and checkpoint blockade) can overcome NK deficits and heighten tumor susceptibility to NK cytotoxicity [159].

Clinical trials using NK cells (Table 3) in recurrent OC have been conducted [160,161,162]. In two phase I studies, ex vivo cultured allogeneic NK cells were administered intraperitoneally, but limitations in cell yield and uncertainty regarding in vivo expansion were observed [161,162]. In a phase II study, Hi-Cy/Flu-based lymphodepletion and IL-2 administration were used to promote NK cell expansion in vivo; however, donor NK cell expansion was inhibited by immunosuppressive factors within the TME. As a result, NK cells were only transiently detectable, and they were largely replaced by host T cells by day 14, indicating a failure of sustained expansion. Additionally, IL-2 administration intended to support NK cell proliferation was associated with concurrent expansion of Tregs [160].

Collectively, preclinical models have shown that OC suppresses NK surveillance through TGF-β1, CISH-linked chronic cytokine/stress programs, and metabolic (iron) conditioning. Convergent solutions—type I IFN restoration (iron chelation), precision CD16a engagement, CIML/CAR engineering (including off-the-shelf products), oncolytic-vIL-2 therapy, and real-time imaging—map a practical route to durable NK-mediated control in the peritoneal cavity [152,153,154]. In clinical studies involving patients with recurrent OC, however, NK cell-based therapies demonstrated limited in vivo expansion and persistence. These findings suggest that, to enhance the efficacy of NK cell-based therapies in the clinic, strategies targeting the TME concurrently with NK cells should be considered [160,161,162].

### 4.6. Cancer-Associated Fibroblasts (CAFs) in Ovarian Cancer Targeting Fibroblast-Driven Oncogenic and Immunosuppressive Pathways

Cancer-associated fibroblasts (CAFs) play key roles within the ovarian cancer microenvironment and have recently emerged as promising therapeutic targets. Multiple studies have reported that CAF-derived growth factors and cytokines directly contribute to OC cell proliferation, invasion, chemoresistance, and the establishment of an immunosuppressive TME [163,164]. Among these factors, CAF-secreted FGF7 promotes OC cell growth and migration and is associated with poor prognosis [164]. In addition, CAF-derived WNT5A drives the maintenance and expansion of cancer stem cells, and inhibition of WNT5A reduces stemness and chemoresistance. These findings indicate that targeting CAF-derived signaling may suppress OC progression and restore responsiveness to anticancer immunotherapies [163].

From an immunotherapy perspective, strategies to target CAFs are also being explored. The fibroblast activation protein-specific 4-1BB ligand fusion protein (FAP-4-1BBL) has shown the ability to enhance intra-tumoral T-cell proliferation and function in preclinical models [165]. Furthermore, early clinical trials of RO7122290, a complex combining FAP binding with 4-1BB co-stimulation, have demonstrated tumor-localized T-cell activation [166]. These results support the concept that CAF-targeted approaches may represent viable immune-enhancing strategies in ovarian cancer.

However, CAFs do not constitute a uniform population; rather, they exhibit substantial heterogeneity, and some CAF subsets can exert tumor-restraining or immunomodulatory effects. Indeed, depletion of specific CAF populations has paradoxically resulted in enhanced metastasis and other unintended consequences, highlighting the potential risks associated with CAF-depletion strategies [167,168]. Moreover, studies directly characterizing the immunoregulatory functions of CAFs in OC remain limited, and a systematic definition of CAF subtypes, cellular origins, and functional markers in this disease is still lacking. Consequently, comprehensive preclinical studies and integrative analyses of CAF heterogeneity are needed to clarify how CAF-derived signals shape immune regulation and to assess the therapeutic potential of targeting CAFs in OC.

## 5. Discussion

**Figure 1:**
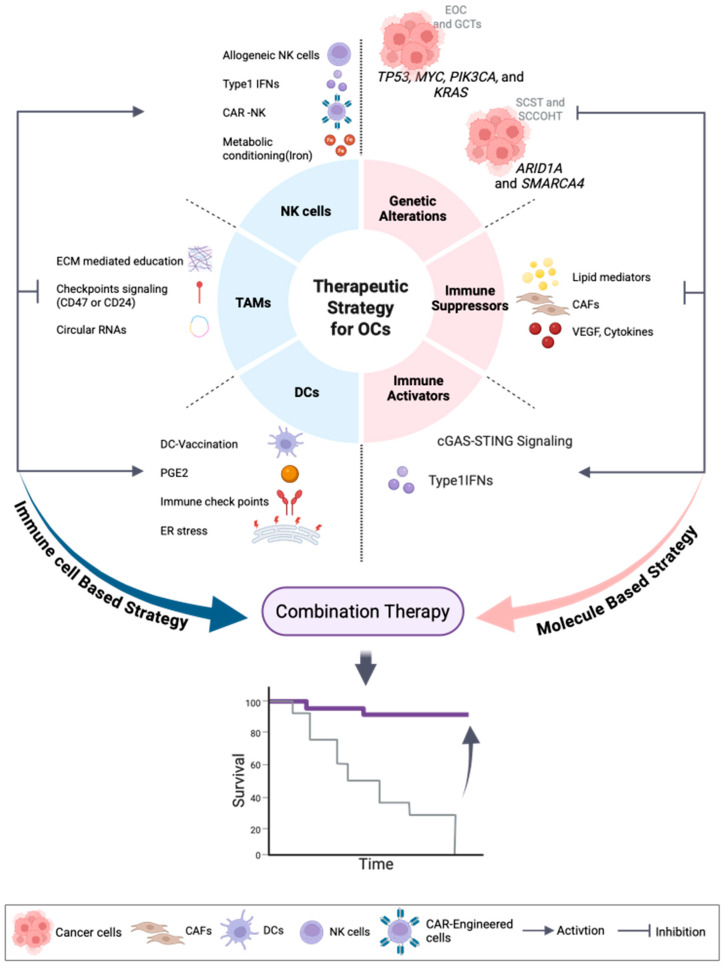
A simplified diagram of combined molecule-based and immune cell-based therapies aimed at improving clinical outcomes. EOCs, epithelial ovarian carcinomas; GCTs, germ cell tumors; SCSTs, sex cord–stromal tumors; SCCOHT, small-cell carcinoma of the ovary, hypercalcemic type; CAF, cancer-associated fibroblast; VEGF, Vascular Endothelial Growth Factor; Type1 IFN, type I interferon; DC, dendritic cell; PGE2, prostaglandin E_2_; ER, endoplasmic reticulum; TAMs, tumor-associated macrophages; ECM, extracellular matrix; NK, natural killer; CAR, Chimeric Antigen Receptor. This image was created by BioRender (http://biorender.com/).

OC is a highly heterogeneous malignancy with diverse histological and molecular subtypes that display distinct clinical behaviors. EOCs constitute the majority, with HGSOC being most common and characterized by TP53 mutations. In contrast, non-epithelial tumors such as SCST and GCT are rarer but harbor unique genetic drivers, including *FOXL2*, *SMARCA4*, and *KRAS*. Despite progress in surgery and chemotherapy, recurrence and drug resistance remain major obstacles. A deeper understanding of subtype-specific molecular alterations and the TME has driven the development of targeted and immune-based therapies. Current therapeutic paradigms are evolving toward multi-modal immune-metabolic approaches that integrate metabolic reprogramming, STING–IFN pathway activation, and myeloid cell (DC and macrophage) reprogramming to convert tumors from “immune-cold” to “immune-active.” Furthermore, engineered NK cells (CAR-NK and CIML-NK) and nanoparticle-based delivery systems enhance immune precision and cytotoxic efficacy. Collectively, these integrated strategies represent a next-generation treatment paradigm aimed at achieving durable immune control and improving long-term survival in OC (Figure 1).

## 6. Conclusions

Despite advances in cytoreductive surgery, platinum-based chemotherapy, and PARP inhibitor-based maintenance therapy, durable control of ovarian cancer remains limited, reflecting gaps in our understanding of oncogenic drivers and tumor–immune interactions. Future progress will require clearer definition of lineage-specific programs and targeted disruption of metabolic and immune regulatory pathways, particularly those shaping lipid-conditioned stromal niches and suppressing type I IFN–STING signaling. Major translational barriers—including intratumoral heterogeneity, poor drug penetration, and resistance to PARP inhibitors and immunotherapy—highlight the need for integrated multiomics profiling, patient-derived models, and improved delivery platforms. Ultimately, therapeutic strategies combining targeted agents with modulators of innate immunity and TME reprogramming hold promise for advancing next-generation ovarian cancer treatment.

## Figures and Tables

**Table 2:** Subtype-specific frequencies of genetic alterations in OCs.

	HGSOC *(n* = 306) [29]	HGSOC (*n* = 42) [108]	HGSOC (*n* = 133) [106]	LGSOC (*n* = 119) [107]	MOC (*n* = 9) [106]	MOC (*n* = 31) [31]	CCC (*n* = 24) [106]	SCST (*n* = 19) [106]	GCT (*n* = 46) [106]	SCCOHT (*n* = 12) [97]
*TP53*	96%	98%	98%	2%	89%	52%	4%		54%	
*Myc*	31%	11%	7%	0%			8%			
*PIK3CA*	18%	4%	9%	<1%	22%		50%			
*SOX2*	15%	0%	4%	0%						
*BRCA1*	12%	7%	2%	0%						
*BRCA2*	12%	13%	1%	<1%						
*NF1*	12%	2%	6%	2%						
*KRAS*	11%	11%	5%	32%	67%	45%	8%		28%	
*SMARCA4*	11%	4%		<1%			8%			92%
*AKT1*	3%	0%	<1%	0%						
*PTEN*	8%	4%	7%	0%					11%	
*CHD4*	8%	─		─						
*BRAF*	7%	0%		9%		23%				
*FOXL2*	5%	0%		0%				68%		
*ARID1A*	2%	4%	7%	<1%		3%	83%			
*CTNNB1*	2%	2%		0%			4%	16%		
*APC*		4%		0%						
*POLE*	2%	0%		0%			4%			
*DICER1*	3%	0%	0%	<1%						
*BCOR*	<1%	7%	4%	<1%			4%			

Genetic-alteration frequencies (%) are cohort-specific. Cohort size and reference for each dataset are provided in the corresponding column. HGSOC, high-grade serous ovarian carcinoma; LGSOC, low-grade serous ovarian carcinoma; MOC, mucinous ovarian carcinoma; CCC, clear cell carcinoma; SCST, sex cord–stromal tumor; GCT, germ cell tumor; SCCOHT, small-cell carcinoma of the ovary, hypercalcemic type.

**Table 3:** Ongoing clinical trials on immune cell-based immune therapy on ClinicalTrials.gov.

Types	NCT Number	Phase	Title	Status	Ref.
DC-based therapy	NCT00799110	2	Vaccination of Patients with Ovarian Cancer with Dendritic Cell/Tumor Fusions with Granulocyte Macrophage Colony-Stimulating Factor (GM-CSF) and Imiquimod	ACTIVE_ NOT_RECRUITING	
	NCT04834544	2	A Study of Maintenance DCVAC/OvCa After First-Line Chemotherapy Added Standard of Care	RECRUITING	
	NCT04739527	1	Phase 1 Study to Evaluate the Safety, Feasibility and Immunogenicity of an Allogeneic, Cell-Based Vaccine (DCP-001) in High-Grade Serous Ovarian Cancer Patients After Primary Treatment	ACTIVE_ NOT_RECRUITING	[142]
	NCT05773859	1/2	NEOadjuvant Dendritic Cell Vaccination for Ovarian Cancer	RECRUITING	[143]
	NCT05920798	1/2	Vaccine Therapy Plus Pembrolizumab in Treating Advanced Ovarian, Fallopian Tube, or Primary Peritoneal Cavity Cancer	RECRUITING	
	NCT05964361	1/2	First-in-Human Interleukin-15-Transpresenting Wilms’ Tumor Protein 1-Targeting Autologous Dendritic Cell Vaccination in Cancer Patients	ACTIVE_ NOT_RECRUITING	
	NCT06639074	2	Folate Receptor Alpha Dendritic Cells or Placebo for the Treatment of Patients with Stage III or IV Ovarian, Fallopian Tube, or Primary Peritoneal Cancer, FAROUT Trial	RECRUITING	
Macrophage-based therapy	NCT01113112	-	Biobehavioral–Cytokine Interactions in Ovarian Cancer	ACTIVE_ NOT_RECRUITING	
	NCT04660929	1	CAR-Macrophages for the Treatment of HER2 Overexpressing Solid Tumors	ACTIVE_ NOT_RECRUITING	[144]
	NCT05053750	Early_ 1	TAME: A Pilot Study of Weekly Paclitaxel, Bevacizumab, and Tumor Associated Macrophage Targeted Therapy (Zoledronic Acid) in Women with Recurrent, Platinum-Resistant, Epithelial Ovarian, Fallopian Tube or Primary Peritoneal Cancer	ACTIVE_ NOT_RECRUITING	
	NCT05467670	2	Safety and Efficacy of Anti-CD47, ALX148 in Combination with Liposomal Doxorubicin and Pembrolizumab in Recurrent Platinum-Resistant Ovarian Cancer	RECRUITING	
	NCT06562647	N.A.	SY001 Targets Mesothelin in a Single-Arm, Dose-Increasing Setting in Subjects with Advanced Solid Tumors	RECRUITING	[145]
	NCT06887673	Early_ 1	Lipid Mediators and Cancer: Montelukast, SPM, and Almonds	NOT_YET_RECRUITING	
NK cell-based therapy	NCT02487693	2	Radiofrequency Ablation Combined with Cytokine-Induced Killer Cells for the Patients with Ovarian Carcinoma	ACTIVE_ NOT_RECRUITING	
	NCT05410717	1	CLDN6/GPC3/Mesothelin/AXL-CAR-NK Cell Therapy for Advanced Solid Tumors	RECRUITING	[146]
	NCT05922930	1/2	Study of TROP2 CAR Engineered IL15-Transduced Cord Blood-derived NK Cells Delivered Intraperitoneally for the Management of Platinum Resistant Ovarian Cancer, Mesonephric-Like Adenocarcinoma, and Pancreatic Cancer	RECRUITING	[147]
	NCT06395844	1/2	Safety and Efficacy of Intraperitoneal Injection of METR-NK Cells as Neoadjuvant Therapy for Advanced Epithelial Ovarian Cancer	RECRUITING	
	NCT06342986	1	Intraperitoneal FT536 in Recurrent Ovarian, Fallopian Tube, and Primary Peritoneal Cancer	RECRUITING	
	NCT05856643	Early_ 1	Single-Arm, Open-Label Clinical Study of SZ011 in the Treatment of Ovarian Epithelial Carcinoma	RECRUITING	
	NCT06321484	1	Intraperitoneal Cytokine-Induced Memory-Like (CIML) Natural Killer (NK) Cells in Recurrent Ovarian Cancer	RECRUITING	
	NCT06884345	1/2	METR-NK Cells in Combination with Anti-Angiogenic Neoadjuvant Therapy for Advanced Epithelial Ovarian Cancer	ACTIVE_ NOT_RECRUITING	
	NCT07096583	1/2	An Exploratory Study on NK Cell-Assisted Prevention of Bone Marrow Suppression During Chemotherapy for Ovarian Cancer	ACTIVE_ NOT_RECRUITING	

Information related to ongoing clinical studies was obtained from https://ClinicalTrials.gov and includes only studies that are currently active, not completed.

## Data Availability

The data that support the findings of this work are available from the corresponding author upon request.

## References

[1] Sherman R.L., Firth A.U., Henley S.J., Siegel R.L., Negoita S., Sung H., Kohler B.A., Anderson R.N., Cucinelli J., Scott S. (2025). Annual Report to the Nation on the Status of Cancer, featuring state-level statistics after the onset of the COVID-19 pandemic. Cancer.

[2] Park E.H., Jung K.W., Park N.J., Kang M.J., Yun E.H., Kim H.J., Kim J.E., Kong H.J., Choi K.S., Yang H.K. (2025). Cancer Statistics in Korea: Incidence, Mortality, Survival, and Prevalence in 2022. Cancer Res. Treat..

[3] Yun B.S., Park E.H., Ha J., Lee J.Y., Lee K.H., Lee T.S., Lee K.J., Kim Y.J., Jung K.W., Roh J.W. (2023). Incidence and survival of gynecologic cancer including cervical, uterine, ovarian, vaginal, vulvar cancer and gestational trophoblastic neoplasia in Korea, 1999-2019: Korea Central Cancer Registry. Obstet. Gynecol. Sci..

[4] Kobel M., Kang E.Y. (2022). The Evolution of Ovarian Carcinoma Subclassification. Cancers.

[5] De Leo A., Santini D., Ceccarelli C., Santandrea G., Palicelli A., Acquaviva G., Chiarucci F., Rosini F., Ravegnini G., Pession A. (2021). What Is New on Ovarian Carcinoma: Integrated Morphologic and Molecular Analysis Following the New 2020 World Health Organization Classification of Female Genital Tumors. Diagnostics.

[6] WHO (2020). Classification of Tumours Editorial Board. Female Genital Tumours.

[7] Wang M., Bi Y., Jin Y., Zheng Z.J. (2024). Global Incidence of Ovarian Cancer According to Histologic Subtype: A Population-Based Cancer Registry Study. JCO Glob. Oncol..

[8] Ha H.I., Chang H.K., Park S.J., Lim J., Won Y.J., Lim M.C. (2021). The incidence and survival of cervical, ovarian, and endometrial cancer in Korea, 1999-2017: Korea Central Cancer Registry. Obstet. Gynecol. Sci..

[9] Matz M., Coleman M.P., Carreira H., Salmeron D., Chirlaque M.D., Allemani C., Group C.W. (2017). Worldwide comparison of ovarian cancer survival: Histological group and stage at diagnosis (CONCORD-2). Gynecol. Oncol..

[10] Peres L.C., Cushing-Haugen K.L., Kobel M., Harris H.R., Berchuck A., Rossing M.A., Schildkraut J.M., Doherty J.A. (2019). Invasive Epithelial Ovarian Cancer Survival by Histotype and Disease Stage. J. Natl. Cancer Inst..

[11] Fortner R.T., Trewin-Nybraten C.B., Paulsen T., Langseth H. (2023). Characterization of ovarian cancer survival by histotype and stage: A nationwide study in Norway. Int. J. Cancer.

[12] De Maria F., Amant F., Chiappa V., Paolini B., Bergamini A., Fruscio R., Corso G., Raspagliesi F., Bogani G. (2025). Malignant germ cells tumor of the ovary. J. Gynecol. Oncol..

[13] Ray-Coquard I., Morice P., Lorusso D., Prat J., Oaknin A., Pautier P., Colombo N., Committee E.G. (2018). Non-epithelial ovarian cancer: ESMO Clinical Practice Guidelines for diagnosis, treatment and follow-up. Ann. Oncol..

[14] Mangili G., Sigismondi C., Gadducci A., Cormio G., Scollo P., Tateo S., Ferrandina G., Greggi S., Candiani M., Lorusso D. (2011). Outcome and risk factors for recurrence in malignant ovarian germ cell tumors: A MITO-9 retrospective study. Int. J. Gynecol. Cancer.

[15] Burdett N.L., Willis M.O., Alsop K., Hunt A.L., Pandey A., Hamilton P.T., Abulez T., Liu X., Hoang T., Craig S. (2023). Multiomic analysis of homologous recombination-deficient end-stage high-grade serous ovarian cancer. Nat. Genet..

[16] Burdett N.L., Willis M.O., Pandey A., Twomey L., Alaei S., Australian Ovarian Cancer Study G., Bowtell D.D.L., Christie E.L. (2024). Timing of whole genome duplication is associated with tumor-specific MHC-II depletion in serous ovarian cancer. Nat. Commun..

[17] Chapel D.B., Joseph N.M., Krausz T., Lastra R.R. (2018). An Ovarian Adenocarcinoma With Combined Low-grade Serous and Mesonephric Morphologies Suggests a Mullerian Origin for Some Mesonephric Carcinomas. Int. J. Gynecol. Pathol..

[18] Cheng E.J., Kurman R.J., Wang M., Oldt R., Wang B.G., Berman D.M., Shih Ie M. (2004). Molecular genetic analysis of ovarian serous cystadenomas. Lab. Investig..

[19] Grisham R.N., Iyer G., Garg K., Delair D., Hyman D.M., Zhou Q., Iasonos A., Berger M.F., Dao F., Spriggs D.R. (2013). BRAF mutation is associated with early stage disease and improved outcome in patients with low-grade serous ovarian cancer. Cancer.

[20] Huang H., Keathley R., Kim U., Cardenas H., Xie P., Wei J., Lengyel E., Nephew K.P., Zhao G., Fu Z. (2024). Comparative transcriptomic, epigenomic and immunological analyses identify drivers of disparity in high-grade serous ovarian cancer. NPJ Genom. Med..

[21] Hunter S.M., Anglesio M.S., Sharma R., Gilks C.B., Melnyk N., Chiew Y.E., deFazio A., Longacre T.A., Huntsman D.G., Australian Ovarian Cancer Study Group (2011). Copy number aberrations in benign serous ovarian tumors: A case for reclassification?. Clin. Cancer Res..

[22] Hussein Y.R., Ducie J.A., Arnold A.G., Kauff N.D., Vargas-Alvarez H.A., Sala E., Levine D.A., Soslow R.A. (2016). Invasion Patterns of Metastatic Extrauterine High-grade Serous Carcinoma With BRCA Germline Mutation and Correlation With Clinical Outcomes. Am. J. Surg. Pathol..

[23] Jones S., Wang T.L., Kurman R.J., Nakayama K., Velculescu V.E., Vogelstein B., Kinzler K.W., Papadopoulos N., Shih Ie M. (2012). Low-grade serous carcinomas of the ovary contain very few point mutations. J. Pathol..

[24] Kelliher L., Yoeli-Bik R., Schweizer L., Lengyel E. (2024). Molecular changes driving low-grade serous ovarian cancer and implications for treatment. Int. J. Gynecol. Cancer.

[25] Malpica A., Wong K.K. (2016). The molecular pathology of ovarian serous borderline tumors. Ann. Oncol..

[26] McCluggage W.G., Vosmikova H., Laco J. (2020). Ovarian Combined Low-grade Serous and Mesonephric-like Adenocarcinoma: Further Evidence for A Mullerian Origin of Mesonephric-like Adenocarcinoma. Int. J. Gynecol. Pathol..

[27] Murali R., Selenica P., Brown D.N., Cheetham R.K., Chandramohan R., Claros N.L., Bouvier N., Cheng D.T., Soslow R.A., Weigelt B. (2019). Somatic genetic alterations in synchronous and metachronous low-grade serous tumours and high-grade carcinomas of the adnexa. Histopathology.

[28] Wong K.K., Tsang Y.T., Deavers M.T., Mok S.C., Zu Z., Sun C., Malpica A., Wolf J.K., Lu K.H., Gershenson D.M. (2010). BRAF mutation is rare in advanced-stage low-grade ovarian serous carcinomas. Am. J. Pathol..

[29] The Cancer Genome Atlas Research Network (2011). Integrated genomic analyses of ovarian carcinoma. Nature.

[30] Veneziani A.C., Gonzalez-Ochoa E., Alqaisi H., Madariaga A., Bhat G., Rouzbahman M., Sneha S., Oza A.M. (2023). Heterogeneity and treatment landscape of ovarian carcinoma. Nat. Rev. Clin. Oncol..

[31] Ryland G.L., Hunter S.M., Doyle M.A., Caramia F., Li J., Rowley S.M., Christie M., Allan P.E., Stephens A.N., Bowtell D.D. (2015). Mutational landscape of mucinous ovarian carcinoma and its neoplastic precursors. Genome. Med..

[32] Seidman J.D., Khedmati F. (2008). Exploring the histogenesis of ovarian mucinous and transitional cell (Brenner) neoplasms and their relationship with Walthard cell nests: A study of 120 tumors. Arch. Pathol. Lab. Med..

[33] Simons M., Simmer F., Bulten J., Ligtenberg M.J., Hollema H., van Vliet S., de Voer R.M., Kamping E.J., van Essen D.F., Ylstra B. (2020). Two types of primary mucinous ovarian tumors can be distinguished based on their origin. Mod. Pathol..

[34] Wu C.H., Mao T.L., Vang R., Ayhan A., Wang T.L., Kurman R.J., Shih Ie M. (2012). Endocervical-type mucinous borderline tumors are related to endometrioid tumors based on mutation and loss of expression of ARID1A. Int. J. Gynecol. Pathol..

[35] McConechy M.K., Ding J., Senz J., Yang W., Melnyk N., Tone A.A., Prentice L.M., Wiegand K.C., McAlpine J.N., Shah S.P. (2014). Ovarian and endometrial endometrioid carcinomas have distinct CTNNB1 and PTEN mutation profiles. Mod. Pathol..

[36] Euscher E.D., Bassett R., Duose D.Y., Lan C., Wistuba I., Ramondetta L., Ramalingam P., Malpica A. (2020). Mesonephric-like Carcinoma of the Endometrium: A Subset of Endometrial Carcinoma With an Aggressive Behavior. Am. J. Surg. Pathol..

[37] Nagamani M., Stuart C.A., Doherty M.G. (1992). Increased steroid production by the ovarian stromal tissue of postmenopausal women with endometrial cancer. J. Clin. Endocrinol. Metab..

[38] Noe M., Ayhan A., Wang T.L., Shih I.M. (2018). Independent development of endometrial epithelium and stroma within the same endometriosis. J. Pathol..

[39] Parra-Herran C., Lerner-Ellis J., Xu B., Khalouei S., Bassiouny D., Cesari M., Ismiil N., Nofech-Mozes S. (2017). Molecular-based classification algorithm for endometrial carcinoma categorizes ovarian endometrioid carcinoma into prognostically significant groups. Mod. Pathol..

[40] Sato N., Tsunoda H., Nishida M., Morishita Y., Takimoto Y., Kubo T., Noguchi M. (2000). Loss of heterozygosity on 10q23.3 and mutation of the tumor suppressor gene PTEN in benign endometrial cyst of the ovary: Possible sequence progression from benign endometrial cyst to endometrioid carcinoma and clear cell carcinoma of the ovary. Cancer Res..

[41] Bennett J.A., Morales-Oyarvide V., Campbell S., Longacre T.A., Oliva E. (2016). Mismatch Repair Protein Expression in Clear Cell Carcinoma of the Ovary: Incidence and Morphologic Associations in 109 Cases. Am. J. Surg. Pathol..

[42] Campbell I.G., Russell S.E., Choong D.Y., Montgomery K.G., Ciavarella M.L., Hooi C.S., Cristiano B.E., Pearson R.B., Phillips W.A. (2004). Mutation of the PIK3CA gene in ovarian and breast cancer. Cancer Res..

[43] Friedlander M.L., Russell K., Millis S., Gatalica Z., Bender R., Voss A. (2016). Molecular Profiling of Clear Cell Ovarian Cancers: Identifying Potential Treatment Targets for Clinical Trials. Int. J. Gynecol. Cancer.

[44] Jones S., Wang T.L., Shih Ie M., Mao T.L., Nakayama K., Roden R., Glas R., Slamon D., Diaz L.A., Vogelstein B. (2010). Frequent mutations of chromatin remodeling gene ARID1A in ovarian clear cell carcinoma. Science.

[45] Karnezis A.N., Wang Y., Ramos P., Hendricks W.P., Oliva E., D’Angelo E., Prat J., Nucci M.R., Nielsen T.O., Chow C. (2016). Dual loss of the SWI/SNF complex ATPases SMARCA4/BRG1 and SMARCA2/BRM is highly sensitive and specific for small cell carcinoma of the ovary, hypercalcaemic type. J. Pathol..

[46] Kuo K.T., Mao T.L., Jones S., Veras E., Ayhan A., Wang T.L., Glas R., Slamon D., Velculescu V.E., Kuman R.J. (2009). Frequent activating mutations of PIK3CA in ovarian clear cell carcinoma. Am. J. Pathol..

[47] Lu F.I., Gilks C.B., Mulligan A.M., Ryan P., Allo G., Sy K., Shaw P.A., Pollett A., Clarke B.A. (2012). Prevalence of loss of expression of DNA mismatch repair proteins in primary epithelial ovarian tumors. Int. J. Gynecol. Pathol..

[48] Parra-Herran C., Bassiouny D., Lerner-Ellis J., Olkhov-Mitsel E., Ismiil N., Hogen L., Vicus D., Nofech-Mozes S. (2019). p53, Mismatch Repair Protein, and POLE Abnormalities in Ovarian Clear Cell Carcinoma: An Outcome-based Clinicopathologic Analysis. Am. J. Surg. Pathol..

[49] Takenaka M., Kobel M., Garsed D.W., Fereday S., Pandey A., Etemadmoghadam D., Hendley J., Kawabata A., Noguchi D., Yanaihara N. (2019). Survival Following Chemotherapy in Ovarian Clear Cell Carcinoma Is Not Associated with Pathological Misclassification of Tumor Histotype. Clin. Cancer Res..

[50] Wang Y.K., Bashashati A., Anglesio M.S., Cochrane D.R., Grewal D.S., Ha G., McPherson A., Horlings H.M., Senz J., Prentice L.M. (2017). Genomic consequences of aberrant DNA repair mechanisms stratify ovarian cancer histotypes. Nat. Genet..

[51] Kurman R.J., Shih Ie M. (2010). The origin and pathogenesis of epithelial ovarian cancer: A proposed unifying theory. Am. J. Surg. Pathol..

[52] Wang T., Huang W., Finkelman B.S., Zhang H. (2024). Malignant Brenner tumor of the ovary: Pathologic evaluation, molecular insights and clinical management. Hum. Pathol. Rep..

[53] Ramphal K., Hadfield M.J., Bandera C.M., Hart J., Dizon D.S. (2023). Genomic and Molecular Characteristics of Ovarian Carcinosarcoma. Am. J. Clin. Oncol..

[54] Mbatani N., Olawaiye A.B., Prat J. (2018). Uterine sarcomas. Int. J. Gynaecol. Obstet..

[55] da Silva E.M., Fix D.J., Sebastiao A.P.M., Selenica P., Ferrando L., Kim S.H., Stylianou A., Da Cruz Paula A., Pareja F., Smith E.S. (2021). Mesonephric and mesonephric-like carcinomas of the female genital tract: Molecular characterization including cases with mixed histology and matched metastases. Mod. Pathol..

[56] Ariyoshi K., Kawauchi S., Kaku T., Nakano H., Tsuneyoshi M. (2000). Prognostic factors in ovarian carcinosarcoma: A clinicopathological and immunohistochemical analysis of 23 cases. Histopathology.

[57] Dasgupta S., Bose D., Bhattacharyya N.K., Biswas P.K. (2015). Carcinosarcoma of ovary with its various immunohistochemical expression: Report of a rare case. J. Cancer Res. Ther..

[58] Jones S., Stransky N., McCord C.L., Cerami E., Lagowski J., Kelly D., Angiuoli S.V., Sausen M., Kann L., Shukla M. (2014). Genomic analyses of gynaecologic carcinosarcomas reveal frequent mutations in chromatin remodelling genes. Nat. Commun..

[59] Sawada M., Tsuda H., Kimura M., Okamoto S., Kita T., Kasamatsu T., Yamada T., Kikuchi Y., Honjo H., Matsubara O. (2003). Different expression patterns of KIT, EGFR, and HER-2 (c-erbB-2) oncoproteins between epithelial and mesenchymal components in uterine carcinosarcoma. Cancer Sci..

[60] Zhao S., Bellone S., Lopez S., Thakral D., Schwab C., English D.P., Black J., Cocco E., Choi J., Zammataro L. (2016). Mutational landscape of uterine and ovarian carcinosarcomas implicates histone genes in epithelial-mesenchymal transition. Proc. Natl. Acad. Sci. USA.

[61] Zheng J., Tang C., Liu P., Hao H. (2023). Carcinosarcoma of the ovary: A case report and literature review. Front. Oncol..

[62] Clement P.B., Scully R.E. (1988). Uterine tumors with mixed epithelial and mesenchymal elements. Semin. Diagn. Pathol..

[63] Liu F.S., Kohler M.F., Marks J.R., Bast R.C., Boyd J., Berchuck A. (1994). Mutation and overexpression of the p53 tumor suppressor gene frequently occurs in uterine and ovarian sarcomas. Obstet. Gynecol..

[64] McCluggage W.G., Singh N., Gilks C.B. (2022). Key changes to the World Health Organization (WHO) classification of female genital tumours introduced in the 5th edition (2020). Histopathology.

[65] Al-Agha O.M., Huwait H.F., Chow C., Yang W., Senz J., Kalloger S.E., Huntsman D.G., Young R.H., Gilks C.B. (2011). FOXL2 is a sensitive and specific marker for sex cord-stromal tumors of the ovary. Am. J. Surg. Pathol..

[66] Buza N., Wong S., Hui P. (2018). FOXL2 Mutation Analysis of Ovarian Sex Cord-Stromal Tumors: Genotype-Phenotype Correlation With Diagnostic Considerations. Int. J. Gynecol. Pathol..

[67] Conlon N., Schultheis A.M., Piscuoglio S., Silva A., Guerra E., Tornos C., Reuter V.E., Soslow R.A., Young R.H., Oliva E. (2015). A survey of DICER1 hotspot mutations in ovarian and testicular sex cord-stromal tumors. Mod. Pathol..

[68] Goulvent T., Ray-Coquard I., Borel S., Haddad V., Devouassoux-Shisheboran M., Vacher-Lavenu M.C., Pujade-Laurraine E., Savina A., Maillet D., Gillet G. (2016). DICER1 and FOXL2 mutations in ovarian sex cord-stromal tumours: A GINECO Group study. Histopathology.

[69] Michal M., Vanecek T., Sima R., Mukensnabl P., Hes O., Kazakov D.V., Matoska J., Zuntova A., Dvorak V., Talerman A. (2006). Mixed germ cell sex cord-stromal tumors of the testis and ovary. Morphological, immunohistochemical, and molecular genetic study of seven cases. Virchows Arch..

[70] de Kock L., Terzic T., McCluggage W.G., Stewart C.J.R., Shaw P., Foulkes W.D., Clarke B.A. (2017). DICER1 Mutations Are Consistently Present in Moderately and Poorly Differentiated Sertoli-Leydig Cell Tumors. Am. J. Surg. Pathol..

[71] Devouassoux-Shisheboran M., Silver S.A., Tavassoli F.A. (1999). Wolffian adnexal tumor, so-called female adnexal tumor of probable Wolffian origin (FATWO): Immunohistochemical evidence in support of a Wolffian origin. Hum. Pathol..

[72] Fumagalli D., Jayraj A., Olearo E., Capasso I., Hsu H.C., Tzur Y., Piedimonte S., Jugeli B., Santana B.N., De Vitis L.A. (2025). Primary versus interval cytoreductive surgery in patients with rare epithelial or non-epithelial ovarian cancer. Int. J. Gynecol. Cancer.

[73] Heravi-Moussavi A., Anglesio M.S., Cheng S.W., Senz J., Yang W., Prentice L., Fejes A.P., Chow C., Tone A., Kalloger S.E. (2012). Recurrent somatic DICER1 mutations in nonepithelial ovarian cancers. N. Engl. J. Med..

[74] Karnezis A.N., Wang Y., Keul J., Tessier-Cloutier B., Magrill J., Kommoss S., Senz J., Yang W., Proctor L., Schmidt D. (2019). DICER1 and FOXL2 Mutation Status Correlates With Clinicopathologic Features in Ovarian Sertoli-Leydig Cell Tumors. Am. J. Surg. Pathol..

[75] Kim S.H., Da Cruz Paula A., Basili T., Dopeso H., Bi R., Pareja F., da Silva E.M., Gularte-Merida R., Sun Z., Fujisawa S. (2020). Identification of recurrent FHL2-GLI2 oncogenic fusion in sclerosing stromal tumors of the ovary. Nat. Commun..

[76] Meurgey A., Descotes F., Mery-Lamarche E., Devouassoux-Shisheboran M. (2017). Lack of mutation of DICER1 and FOXL2 genes in microcystic stromal tumor of the ovary. Virchows Arch..

[77] Schultz K.A.P., Harris A.K., Finch M., Dehner L.P., Brown J.B., Gershenson D.M., Young R.H., Field A., Yu W., Turner J. (2017). DICER1-related Sertoli-Leydig cell tumor and gynandroblastoma: Clinical and genetic findings from the International Ovarian and Testicular Stromal Tumor Registry. Gynecol. Oncol..

[78] Tchrakian N., Oliva E., Chong A.S., Rivera-Polo B., Bennett J.A., Nucci M.R., Sah S., Schoolmeester J.K., van der Griend R.A., Foulkes W.D. (2022). Ovarian Signet-ring Stromal Tumor: A Morphologic, Immunohistochemical, and Molecular Study of 7 Cases With Discussion of the Differential Diagnosis. Am. J. Surg. Pathol..

[79] Zhang Y., Tao L., Yin C., Wang W., Zou H., Ren Y., Liang W., Jiang J., Zhang W., Jia W. (2018). Ovarian microcystic stromal tumor with undetermined potential: Case study with molecular analysis and literature review. Hum. Pathol..

[80] Rabban J.T., Karnezis A.N., Devine W.P. (2020). Practical roles for molecular diagnostic testing in ovarian adult granulosa cell tumour, Sertoli-Leydig cell tumour, microcystic stromal tumour and their mimics. Histopathology.

[81] Schmidt J., Derr V., Heinrich M.C., Crum C.P., Fletcher J.A., Corless C.L., Nose V. (2007). BRAF in papillary thyroid carcinoma of ovary (struma ovarii). Am. J. Surg. Pathol..

[82] Cools M., Looijenga L.H., Wolffenbuttel K.P., Drop S.L. (2009). Disorders of sex development: Update on the genetic background, terminology and risk for the development of germ cell tumors. World J. Pediatr..

[83] Riopel M.A., Spellerberg A., Griffin C.A., Perlman E.J. (1998). Genetic analysis of ovarian germ cell tumors by comparative genomic hybridization. Cancer Res..

[84] Stoop H., Honecker F., van de Geijn G.J., Gillis A.J., Cools M.C., de Boer M., Bokemeyer C., Wolffenbuttel K.P., Drop S.L., de Krijger R.R. (2008). Stem cell factor as a novel diagnostic marker for early malignant germ cells. J. Pathol..

[85] Cheng L., Roth L.M., Zhang S., Wang M., Morton M.J., Zheng W., Abdul Karim F.W., Montironi R., Lopez-Beltran A. (2011). KIT gene mutation and amplification in dysgerminoma of the ovary. Cancer.

[86] Cheng L., Zhang S., Talerman A., Roth L.M. (2010). Morphologic, immunohistochemical, and fluorescence in situ hybridization study of ovarian embryonal carcinoma with comparison to solid variant of yolk sac tumor and immature teratoma. Hum. Pathol..

[87] Cossu-Rocca P., Zhang S., Roth L.M., Eble J.N., Zheng W., Karim F.W., Michael H., Emerson R.E., Jones T.D., Hattab E.M. (2006). Chromosome 12p abnormalities in dysgerminoma of the ovary: A FISH analysis. Mod. Pathol..

[88] Hersmus R., Kalfa N., de Leeuw B., Stoop H., Oosterhuis J.W., de Krijger R., Wolffenbuttel K.P., Drop S.L., Veitia R.A., Fellous M. (2008). FOXL2 and SOX9 as parameters of female and male gonadal differentiation in patients with various forms of disorders of sex development (DSD). J. Pathol..

[89] Liang L., Zhang Y., Malpica A., Ramalingam P., Euscher E.D., Fuller G.N., Liu J. (2015). Gliomatosis peritonei: A clinicopathologic and immunohistochemical study of 21 cases. Mod. Pathol..

[90] Poulos C., Cheng L., Zhang S., Gersell D.J., Ulbright T.M. (2006). Analysis of ovarian teratomas for isochromosome 12p: Evidence supporting a dual histogenetic pathway for teratomatous elements. Mod. Pathol..

[91] Snir O.L., DeJoseph M., Wong S., Buza N., Hui P. (2017). Frequent homozygosity in both mature and immature ovarian teratomas: A shared genetic basis of tumorigenesis. Mod. Pathol..

[92] Witkowski L., Mattina J., Schonberger S., Murray M.J., Choong C.S., Huntsman D.G., Reis-Filho J.S., McCluggage W.G., Nicholson J.C., Coleman N. (2013). DICER1 hotspot mutations in non-epithelial gonadal tumours. Br. J. Cancer.

[93] Kupryjanczyk J., Dansonka-Mieszkowska A., Moes-Sosnowska J., Plisiecka-Halasa J., Szafron L., Podgorska A., Rzepecka I.K., Konopka B., Budzilowska A., Rembiszewska A. (2013). Ovarian small cell carcinoma of hypercalcemic type—Evidence of germline origin and SMARCA4 gene inactivation. a pilot study. Pol. J. Pathol..

[94] Ramachandra C.A., Attili V.S.S., Dadhich H.P., Kumari A., Appaji L., Giri G.V., Mukharjee G. (2007). Extrarenal Wilms’ tumor: A report of two cases and review of literature. J. Indian Assoc. Pediatr. Surg..

[95] Kominami A., Fujino M., Murakami H., Ito M. (2014). beta-catenin mutation in ovarian solid pseudopapillary neoplasm. Pathol. Int..

[96] Singh K., Patel N., Patil P., Paquette C., Mathews C.A., Lawrence W.D. (2018). Primary Ovarian Solid Pseudopapillary Neoplasm With CTNNB1 c.98C>G (p.S33C) Point Mutation. Int. J. Gynecol. Pathol..

[97] Jelinic P., Mueller J.J., Olvera N., Dao F., Scott S.N., Shah R., Gao J., Schultz N., Gonen M., Soslow R.A. (2014). Recurrent SMARCA4 mutations in small cell carcinoma of the ovary. Nat. Genet..

[98] Ramos P., Karnezis A.N., Craig D.W., Sekulic A., Russell M.L., Hendricks W.P., Corneveaux J.J., Barrett M.T., Shumansky K., Yang Y. (2014). Small cell carcinoma of the ovary, hypercalcemic type, displays frequent inactivating germline and somatic mutations in SMARCA4. Nat. Genet..

[99] Witkowski L., Carrot-Zhang J., Albrecht S., Fahiminiya S., Hamel N., Tomiak E., Grynspan D., Saloustros E., Nadaf J., Rivera B. (2014). Germline and somatic SMARCA4 mutations characterize small cell carcinoma of the ovary, hypercalcemic type. Nat. Genet..

[100] Fahiminiya S., Witkowski L., Nadaf J., Carrot-Zhang J., Goudie C., Hasselblatt M., Johann P., Kool M., Lee R.S., Gayden T. (2016). Molecular analyses reveal close similarities between small cell carcinoma of the ovary, hypercalcemic type and atypical teratoid/rhabdoid tumor. Oncotarget.

[101] Witkowski L., Goudie C., Ramos P., Boshari T., Brunet J.S., Karnezis A.N., Longy M., Knost J.A., Saloustros E., McCluggage W.G. (2016). The influence of clinical and genetic factors on patient outcome in small cell carcinoma of the ovary, hypercalcemic type. Gynecol. Oncol..

[102] Holdhof D., Johann P.D., Spohn M., Bockmayr M., Safaei S., Joshi P., Masliah-Planchon J., Ho B., Andrianteranagna M., Bourdeaut F. (2021). Atypical teratoid/rhabdoid tumors (ATRTs) with SMARCA4 mutation are molecularly distinct from SMARCB1-deficient cases. Acta Neuropathol..

[103] Kommoss F.K., Tessier-Cloutier B., Witkowski L., Forgo E., Koelsche C., Kobel M., Foulkes W.D., Lee C.H., Kolin D.L., von Deimling A. (2022). Cellular context determines DNA methylation profiles in SWI/SNF-deficient cancers of the gynecologic tract. J. Pathol..

[104] Ben-Rafael Z., Bider D., Menashe Y., Maymon R., Zolti M., Mashiach S. (1990). Follicular and luteal cysts after treatment with gonadotropin-releasing hormone analog for in vitro fertilization. Fertil. Steril..

[105] Bailey M.H., Tokheim C., Porta-Pardo E., Sengupta S., Bertrand D., Weerasinghe A., Colaprico A., Wendl M.C., Kim J., Reardon B. (2018). Comprehensive Characterization of Cancer Driver Genes and Mutations. Cell.

[106] Zehir A., Benayed R., Shah R.H., Syed A., Middha S., Kim H.R., Srinivasan P., Gao J., Chakravarty D., Devlin S.M. (2017). Mutational landscape of metastatic cancer revealed from prospective clinical sequencing of 10,000 patients. Nat. Med..

[107] Manning-Geist B., Gordhandas S., Liu Y.L., Zhou Q., Iasonos A., Da Cruz Paula A., Mandelker D., Long Roche K., Zivanovic O., Maio A. (2022). MAPK Pathway Genetic Alterations Are Associated with Prolonged Overall Survival in Low-Grade Serous Ovarian Carcinoma. Clin. Cancer Res..

[108] Elkin R., Oh J.H., Liu Y.L., Selenica P., Weigelt B., Reis-Filho J.S., Zamarin D., Deasy J.O., Norton L., Levine A.J. (2021). Geometric network analysis provides prognostic information in patients with high grade serous carcinoma of the ovary treated with immune checkpoint inhibitors. NPJ Genom. Med..

[109] Matuszczak M., Kiljanczyk A., Marciniak W., Derkacz R., Stempa K., Baszuk P., Bryskiewicz M., Cybulski C., Debniak T., Jacek G. (2024). Blood molybdenum level as a marker of cancer risk on BRCA1 carriers. Hered. Cancer Clin. Pract..

[110] Singh K.A., Soukar J., Zulkifli M., Kersey A., Lokhande G., Ghosh S., Murali A., Garza N.M., Kaur H., Keeney J.N. (2024). Atomic vacancies of molybdenum disulfide nanoparticles stimulate mitochondrial biogenesis. Nat. Commun..

[111] Giornelli G.H. (2016). Management of relapsed ovarian cancer: A review. Springerplus.

[112] Siegel R.L., Miller K.D., Jemal A. (2016). Cancer statistics, 2016. CA Cancer J. Clin..

[113] Ray-Coquard I., Leary A., Pignata S., Cropet C., Gonzalez-Martin A., Marth C., Nagao S., Vergote I., Colombo N., Maenpaa J. (2023). Olaparib plus bevacizumab first-line maintenance in ovarian cancer: Final overall survival results from the PAOLA-1/ENGOT-ov25 trial. Ann. Oncol..

[114] DiSilvestro P., Banerjee S., Colombo N., Scambia G., Kim B.G., Oaknin A., Friedlander M., Lisyanskaya A., Floquet A., Leary A. (2023). Overall Survival With Maintenance Olaparib at a 7-Year Follow-Up in Patients With Newly Diagnosed Advanced Ovarian Cancer and a BRCA Mutation: The SOLO1/GOG 3004 Trial. J. Clin. Oncol..

[115] Cuenca-Escalona J., Bodder J., Subtil B., Sanchez-Sanchez M., Vidal-Manrique M., Sweep M.W.D., Fauerbach J.A., Cambi A., Florez-Grau G., de Vries J.M. (2024). EP2/EP4 targeting prevents tumor-derived PGE2-mediated immunosuppression in cDC2s. J. Leukoc. Biol..

[116] Chaudhry S., Thomas S.N., Simmons G.E. (2022). Targeting lipid metabolism in the treatment of ovarian cancer. Oncotarget.

[117] Surendran A., Jamalkhah M., Poutou J., Birtch R., Lawson C., Dave J., Crupi M.J.F., Mayer J., Taylor V., Petryk J. (2023). Fatty acid transport protein inhibition sensitizes breast and ovarian cancers to oncolytic virus therapy via lipid modulation of the tumor microenvironment. Front. Immunol..

[118] Chen R.R., Yung M.M.H., Xuan Y., Zhan S., Leung L.L., Liang R.R., Leung T.H.Y., Yang H., Xu D., Sharma R. (2025). Author Correction: Targeting of lipid metabolism with a metabolic inhibitor cocktail eradicates peritoneal metastases in ovarian cancer cells. Commun. Biol..

[119] He Z.Y., Deng F., Wei X.W., Ma C.C., Luo M., Zhang P., Sang Y.X., Liang X., Liu L., Qin H.X. (2016). Ovarian cancer treatment with a tumor-targeting and gene expression-controllable lipoplex. Sci. Rep..

[120] Zhang D., Zhang B. (2025). cGAS/STING signaling pathway in gynecological malignancies: From molecular mechanisms to therapeutic values. Front. Immunol..

[121] Ding L., Kim H.J., Wang Q., Kearns M., Jiang T., Ohlson C.E., Li B.B., Xie S., Liu J.F., Stover E.H. (2018). PARP Inhibition Elicits STING-Dependent Antitumor Immunity in Brca1-Deficient Ovarian Cancer. Cell Rep..

[122] Meng J., Peng J., Feng J., Maurer J., Li X., Li Y., Yao S., Chu R., Pan X., Li J. (2021). Niraparib exhibits a synergistic anti-tumor effect with PD-L1 blockade by inducing an immune response in ovarian cancer. J. Transl. Med..

[123] Cornelison R., Biswas K., Llaneza D.C., Harris A.R., Sosale N.G., Lazzara M.J., Landen C.N. (2021). CX-5461 Treatment Leads to Cytosolic DNA-Mediated STING Activation in Ovarian Cancer. Cancers.

[124] Zhang J., Chen Y., Chen X., Zhang W., Zhao L., Weng L., Tian H., Wu Z., Tan X., Ge X. (2021). Deubiquitinase USP35 restrains STING-mediated interferon signaling in ovarian cancer. Cell Death Differ..

[125] Xie W., Zhang L., Shen J., Lai F., Han W., Liu X. (2024). Knockdown of CENPM activates cGAS-STING pathway to inhibit ovarian cancer by promoting pyroptosis. BMC Cancer.

[126] Xu Y., Huang X., Nie X.C., Liu Y.S., Zhou Y., Niu J.M. (2024). Manganese and IL-12 treatment alters the ovarian tumor microenvironment. Aging.

[127] Shen J., Xu F., Liu T., Ye Y., Xu S. (2025). NAD(+) Metabolism-Mediated SURF4-STING Axis Enhances T-Cell Anti-Tumor Effects in the Ovarian Cancer Microenvironment. Cell Death Dis..

[128] Zhang H., Sun Y., Zhang L., Shen M., Tong X., Zhao D., Xiao H., Li B. (2025). Polymer-PARPi Conjugates Delivering USP1i for Maximizing Synthetic Lethality to Stimulate STING Pathway in High-Grade Serous Ovarian Cancer. Adv. Mater..

[129] Chae C.S., Teran-Cabanillas E., Cubillos-Ruiz J.R. (2017). Dendritic cell rehab: New strategies to unleash therapeutic immunity in ovarian cancer. Cancer Immunol. Immunother..

[130] Galpin K.J.C., Rodriguez G.M., Maranda V., Cook D.P., Macdonald E., Murshed H., Zhao S., McCloskey C.W., Chruscinski A., Levy G.A. (2024). FGL2 promotes tumour growth and attenuates infiltration of activated immune cells in melanoma and ovarian cancer models. Sci. Rep..

[131] Zhang X., He T., Li Y., Chen L., Liu H., Wu Y., Guo H. (2020). Dendritic Cell Vaccines in Ovarian Cancer. Front. Immunol..

[132] Caro A.A., Deschoemaeker S., Allonsius L., Coosemans A., Laoui D. (2022). Dendritic Cell Vaccines: A Promising Approach in the Fight against Ovarian Cancer. Cancers.

[133] Zhang L., Zhao W., Huang J., Li F., Sheng J., Song H., Chen Y. (2022). Development of a Dendritic Cell/Tumor Cell Fusion Cell Membrane Nano-Vaccine for the Treatment of Ovarian Cancer. Front. Immunol..

[134] Zhang X., Tang D., Xiao H., Li B., Shang K., Zhao D. (2025). Activating the cGAS-STING Pathway by Manganese-Based Nanoparticles Combined with Platinum-Based Nanoparticles for Enhanced Ovarian Cancer Immunotherapy. ACS Nano.

[135] Puttock E.H., Tyler E.J., Manni M., Maniati E., Butterworth C., Burger Ramos M., Peerani E., Hirani P., Gauthier V., Liu Y. (2023). Extracellular matrix educates an immunoregulatory tumor macrophage phenotype found in ovarian cancer metastasis. Nat. Commun..

[136] Fang J., Wang J., Zhao X., Yang Y., Xiao Y. (2025). KLHDC8A knockdown in normal ovarian epithelial cells promoted the polarization of pro-tumoral macrophages via the C5a/C5aR/p65 NFκB signaling pathway. Cell Immunol..

[137] Luan X., Lei T., Fang J., Liu X., Fu H., Li Y., Chu W., Jiang P., Tong C., Qi H. (2024). Blockade of C5a receptor unleashes tumor-associated macrophage antitumor response and enhances CXCL9-dependent CD8(+) T cell activity. Mol. Ther..

[138] Liu R., Wei H., Gao P., Yu H., Wang K., Fu Z., Ju B., Zhao M., Dong S., Li Z. (2017). CD47 promotes ovarian cancer progression by inhibiting macrophage phagocytosis. Oncotarget.

[139] Yang Y., Wu H., Yang Y., Kang Y., He R., Zhou B., Guo H., Zhang J., Li J., Ge C. (2023). Dual blockade of CD47 and CD24 signaling using a novel bispecific antibody fusion protein enhances macrophage immunotherapy. Mol. Ther. Oncolytics.

[140] Lakhani N.J., Stewart D., Richardson D.L., Dockery L.E., Van Le L., Call J., Rangwala F., Wang G., Ma B., Metenou S. (2025). First-in-human phase I trial of the bispecific CD47 inhibitor and CD40 agonist Fc-fusion protein, SL-172154 in patients with platinum-resistant ovarian cancer. J. Immunother. Cancer.

[141] Li H., Luo F., Jiang X., Zhang W., Xiang T., Pan Q., Cai L., Zhao J., Weng D., Li Y. (2022). CircITGB6 promotes ovarian cancer cisplatin resistance by resetting tumor-associated macrophage polarization toward the M2 phenotype. J. Immunother. Cancer.

[142] Vledder A., Zeeburg H.v., Brummel K., Eerkens A.L., Rooij N.v., Plat A., Rovers J., Bruyn M.d., Nijman H. (2025). Successful induction of tumor-directed immune responses in high grade serious ovarian carcinoma patients after primary treatment using a whole tumor cell vaccine. J. Clin. Oncol..

[143] Koeneman B., Schreibelt G., Duiveman-de Boer T., Bos K., van Oorschot T., Pots J., Scharenborg N., de Boer A., Hins-de Bree S., de Haas N. (2025). NEOadjuvant Dendritic cell therapy added to first line standard of care in advanced epithelial Ovarian Cancer (NEODOC): Protocol of a first-in-human, exploratory, single-centre phase I/II trial in the Netherlands. BMJ Open.

[144] Reiss K.A., Angelos M.G., Dees E.C., Yuan Y., Ueno N.T., Pohlmann P.R., Johnson M.L., Chao J., Shestova O., Serody J.S. (2025). CAR-macrophage therapy for HER2-overexpressing advanced solid tumors: A phase 1 trial. Nat. Med..

[145] Li X., Wang X., Wang H., Zuo D., Xu J., Feng Y., Xue D., Zhang L., Lin L., Zhang J. (2024). A clinical study of autologous chimeric antigen receptor macrophage targeting mesothelin shows safety in ovarian cancer therapy. J. Hematol. Oncol..

[146] Li J., Hu H., Lian H., Yang S., Liu M., He J., Cao B., Chen D., Hu Y., Zhi C. (2024). NK-92MI Cells Engineered with Anti-claudin-6 Chimeric Antigen Receptors in Immunotherapy for Ovarian Cancer. Int. J. Biol. Sci..

[147] Knisely A., Rafei H., Basar R., Banerjee P.P., Metwalli Z., Lito K., Fellman B.M., Yuan Y., Wolff R.A., Morelli M.P. (2024). Phase I/II study of TROP2 CAR engineered IL15-transduced cord blood-derived NK cells delivered intraperitoneally for the management of platinum resistant ovarian cancer, mesonephric-like adenocarcinoma, and pancreatic cancer. J. Clin. Oncol..

[148] VandenHeuvel S.N., Chau E., Mohapatra A., Dabbiru S., Roy S., O’Connell C., Kamat A., Godin B., Raghavan S.A. (2024). Macrophage Checkpoint Nanoimmunotherapy Has the Potential to Reduce Malignant Progression in Bioengineered In Vitro Models of Ovarian Cancer. ACS Appl. Bio. Mater..

[149] Liu Y., Yang J., Hilliard T.S., Wang Z., Johnson J., Wang W., Harper E.I., Ott C., O’Brien C., Campbell L. (2023). Host obesity alters the ovarian tumor immune microenvironment and impacts response to standard of care chemotherapy. J. Exp. Clin. Cancer Res..

[150] Luke J.J., Barlesi F., Chung K., Tolcher A.W., Kelly K., Hollebecque A., Le Tourneau C., Subbiah V., Tsai F., Kao S. (2021). Phase I study of ABBV-428, a mesothelin-CD40 bispecific, in patients with advanced solid tumors. J. Immunother. Cancer.

[151] Yeku O.O., Barve M., Tan W.W., Wang J., Patnaik A., LoRusso P., Richardson D.L., Naqash A.R., Lynam S.K., Fu S. (2025). Myeloid targeting antibodies PY159 and PY314 for platinum-resistant ovarian cancer. J. Immunother. Cancer.

[152] Maas R.J.A., Hoogstad-van Evert J.S., Hagemans I.M., Brummelman J., van Ens D., de Jonge P., Hooijmaijers L., Mahajan S., van der Waart A.B., Hermans C. (2024). Increased peritoneal TGF-beta1 is associated with ascites-induced NK-cell dysfunction and reduced survival in high-grade epithelial ovarian cancer. Front. Immunol..

[153] Acosta J.C., Bahr J.M., Basu S., O’Donnell J.T., Barua A. (2023). Expression of CISH, an Inhibitor of NK Cell Function, Increases in Association with Ovarian Cancer Development and Progression. Biomedicines.

[154] Sandoval T.A., Salvagno C., Chae C.S., Awasthi D., Giovanelli P., Marin Falco M., Hwang S.M., Teran-Cabanillas E., Suominen L., Yamazaki T. (2024). Iron Chelation Therapy Elicits Innate Immune Control of Metastatic Ovarian Cancer. Cancer Discov..

[155] Tarannum M.A.-O., Dinh K.A.-O.X., Vergara J.A.-O., Birch G., Abdulhamid Y.A.-O., Kaplan I.A.-O., Ay O., Maia A.A.-O., Beaver O., Sheffer M.A.-O. (2024). CAR memory-like NK cells targeting the membrane proximal domain of mesothelin demonstrate promising activity in ovarian cancer. Sci. Adv..

[156] Liu Y., Zhang M., Shen X., Xia C., Hu F., Huang D., Weng Q., Zhang Q., Liu L., Zhu Y. (2024). Mesothelin CAR-engineered NK cells derived from human embryonic stem cells suppress the progression of human ovarian cancer in animals. Cell Prolif..

[157] Shalaby N., Xia Y., Kelly J.J., Sanchez-Pupo R., Martinez F., Fox M.S., Thiessen J.D., Hicks J.W., Scholl T.J., Ronald J.A. (2024). Imaging CAR-NK cells targeted to HER2 ovarian cancer with human sodium-iodide symporter-based positron emission tomography. Eur. J. Nucl. Med. Mol. Imaging.

[158] Quixabeira D.C.A., Pakola S., Jirovec E., Havunen R., Basnet S., Santos J.M., Kudling T.V., Clubb J.H.A., Haybout L., Arias V. (2023). Boosting cytotoxicity of adoptive allogeneic NK cell therapy with an oncolytic adenovirus encoding a human vIL-2 cytokine for the treatment of human ovarian cancer. Cancer Gene Ther..

[159] Hou Y., Zhao X., Nie X. (2024). Enhancing the therapeutic efficacy of NK cells in the treatment of ovarian cancer (Review). Oncol. Rep..

[160] Geller M.A., Cooley S., Judson P.L., Ghebre R., Carson L.F., Argenta P.A., Jonson A.L., Panoskaltsis-Mortari A., Curtsinger J., McKenna D. (2011). A phase II study of allogeneic natural killer cell therapy to treat patients with recurrent ovarian and breast cancer. Cytotherapy.

[161] Hoogstad-van Evert J., Bekkers R., Ottevanger N., Schaap N., Hobo W., Jansen J.H., Massuger L., Dolstra H. (2019). Intraperitoneal infusion of ex vivo-cultured allogeneic NK cells in recurrent ovarian carcinoma patients (a phase I study). Medicine.

[162] Evert J.S.H., de Jonge P., Zusterzeel P.L.M., Hobo W., van der Waart A.B., Fredrix H., Janssen L., Wuts M., Bosmans L., Spijkers E. (2025). Intraperitoneal infusion of stem cell-derived natural killer cells in recurrent epithelial ovarian cancer patients: Results of the phase 1 INTRO-01 trial. Gynecol. Oncol..

[163] Fang Y., Xiao X., Wang J., Dasari S., Pepin D., Nephew K.P., Zamarin D., Mitra A.K. (2024). Cancer associated fibroblasts serve as an ovarian cancer stem cell niche through noncanonical Wnt5a signaling. NPJ Precis. Oncol..

[164] Feng S., Ding B., Dai Z., Yin H., Ding Y., Liu S., Zhang K., Lin H., Xiao Z., Shen Y. (2024). Cancer-associated fibroblast-secreted FGF7 as an ovarian cancer progression promoter. J. Transl. Med..

[165] Trub M., Uhlenbrock F., Claus C., Herzig P., Thelen M., Karanikas V., Bacac M., Amann M., Albrecht R., Ferrara-Koller C. (2020). Fibroblast activation protein-targeted-4-1BB ligand agonist amplifies effector functions of intratumoral T cells in human cancer. J. Immunother. Cancer.

[166] Melero I., Tanos T., Bustamante M., Sanmamed M.F., Calvo E., Moreno I., Moreno V., Hernandez T., Martinez Garcia M., Rodriguez-Vida A. (2023). A first-in-human study of the fibroblast activation protein-targeted, 4-1BB agonist RO7122290 in patients with advanced solid tumors. Sci. Transl. Med..

[167] Li Y., Liu Q., Jing X., Wang Y., Jia X., Yang X., Chen K. (2025). Cancer-Associated Fibroblasts: Heterogeneity, Cancer Pathogenesis, and Therapeutic Targets. MedComm.

[168] Rupert J., Daquinag A., Yu Y., Dai Y., Zhao Z., Kolonin M.G. (2025). Depletion of Adipose Stroma-Like Cancer-Associated Fibroblasts Potentiates Pancreatic Cancer Immunotherapy. Cancer Res. Commun..

